# New insights into the molecular mechanism of the Rab GTPase Sec4p activation

**DOI:** 10.1186/s12900-015-0041-5

**Published:** 2015-08-12

**Authors:** Fabio C. Rinaldi, Michael Packer, Ruth Collins

**Affiliations:** Department of Molecular Medicine, Cornell University, Ithaca, NY 14853 USA; Honors Program in Undergraduate Studies, Cornell University, Ithaca, NY 14853 USA

## Abstract

**Background:**

Sec4p is a small monomeric Ras-related GTP-binding protein (23 kDa) that regulates polarized exocytosis in *S. cerevisiae*. In this study we examine the structural effects of a conserved serine residue in the P-loop corresponding to G12 in Ras.

**Results:**

We show that the Sec4p residue serine 29 forms a hydrogen bond with the nucleotide. Mutations of this residue have a different impact than equivalent mutations in Ras and can form stable associations with the exchange factor allowing us to elucidate the structure of a complex of Sec4p bound to the exchange factor Sec2p representing an early stage of the exchange reaction.

**Conclusions:**

Our structural investigation of the Sec4p-Sec2p complex reveals the role of the Sec2p coiled-coil domain in facilitating the fast kinetics of the exchange reaction. For Ras-family GTPases, single point mutations that impact the signaling state of the molecule have been well described however less structural information is available for equivalent mutations in the case of Rab proteins. Understanding the structural properties of mutants such as the one described here, provides useful insights into unique aspects of Rab GTPase function.

## Background

Rab GTPases comprise the largest family of proteins among the small Ras-like GTPase superfamily functioning as critical regulators of a variety of membrane transport processes [1]. Sec4p is among the 11 Rab GTPases identified in *S. cerevisiae* and acts on the surface of membranes to regulate transport between the Golgi apparatus and the plasma membrane [2–4]. The localization pattern of Sec4p depends upon its guanine nucleotide bound state. In the GTP bound state Sec4p exhibits an active conformation capable of interaction with targets on the plasma membrane. After GTP hydrolysis, the protein becomes inactive for effector recruitment and is then recycled from the plasma membrane and delivered back to the donor membrane. To complete the cycle, the protein is reactivated by switching the guanine nucleotide state from GDP to GTP in a process stimulated by guanine nucleotide exchange factors (GEFs). The GTPase cycle is controlled by interactions with regulators and effectors and is dependent on structural changes caused by the guanine nucleotide bound state of the protein [5–8]. Members of the GTPase family are known to share high overall structural homology. However, the differences in sequence identity of key domains known as Switch I, Switch II and the P-loop are sufficient to establish the interaction with specific regulators and effectors [5, 9–12]. Mutations in equivalent regions in Ras are commonly present in oncogenic cells and are known to perturb interactions with regulators leading to imbalance of the GTPase cycle. For example, Ras mutation Gly12Val, which is located in the P-loop region, confers reduced intrinsic GTPase activity and insensitivity to the action of GTPase Activating Protein (GAP) [13]. As a result, this Ras variant preferentially remains in the GTP-bound conformation leading to constitutive signaling and cell transformation [14, 15].

**Fig. 1:**
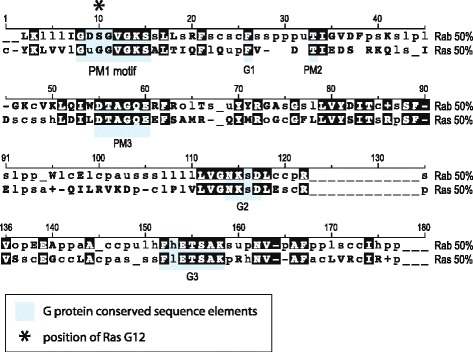
Comparison of the core domain of Ras superfamily sequences between Rab, Ras and Rho subfamilies. The core domain is aligned showing in uppercase bold, those residues conserved at the 50 % consensus level i.e. at least 50 % sequences show this residue at the position indicated. Bold is also used for positions conserved for positive (+, H, K, R) or negative charge (−, D, E). In lowercase is shown the consensus sequence at non-conserved positions designated according to the amino acid class abbreviation; o (alcohol, S,T), l (aliphatic (I, L,V), a (aromatic, F, H,W,Y), c (charged, D,E,H,K,R), h (hydrophobic, A,C,F,G,H,I,K,L,M,R,T,V,W,Y), p (polar, C,D,E,H,K,N,Q,R,S,T), s (small, A,C,D,G,N,P,S,T,V), u (tiny, A,G,S), t (turn-like, A,C,D,E,G,H,K,N,Q,R,S,T). All residues that are conserved at the 50 % consensus level between the Ras and Rab families are shaded in black. Consensus sequence data were obtained from the SMART database (http://smart.embl-heidelberg.de/) [40] and are derived from 339 Ras domains and 1120 Rab domains. For greater clarification, the G protein conserved sequence elements are shown highlighted in grey. Numbering is arbitrary and intended as a descriptive guide. An asterisk marks the Ras glycine 12 position, this is highly conserved amongst Ras family members and the equivalent residue in at least 50 % of Rab proteins is serine

The residue equivalent to RasG12 in Rab proteins is a serine that is well conserved throughout the Rab GTPase family (Fig. 1). Interestingly mutations in either the P-loop serine for Rab GTPases (Ser29 for Sec4p) or Gly12 for Ras affect cell growth and result in completely opposite phenotypes. RasG12V results in a GTP bound state (active state), whereas for Rab proteins the mutant serine to valine has been observed to abolish sensitivity to GEF activation by locking the protein in the GDP bound state (inactive state) [9, 14, 16]. Thus, although structurally equivalent, this site provides different functions in these orthologous proteins by influencing interactions with their respective regulators and the bound nucleotides. Although the G12V Ras mutant has been the focus of extensive research, little is known about the equivalent mutant in Rabs. Therefore, the structural and functional analysis of the mutant Ser29Val of Sec4p offers an opportunity to understand not just the basic differences between Ras GTPase families but also how Sec4p is activated during its GTPase cycle by its GEF Sec2p. Sec2p is a highly efficient exchange factor of Sec4p that stimulates nucleotide exchange by binding to the switch regions of Sec4p and altering its conformation resulting in decreased affinity between Sec4p and nucleotide [5, 17].

In this study we investigate the activation mechanism of Sec4p by making use of the Ser29Val mutant. Both intrinsic and stimulated nucleotide exchange reactions of Sec4p^V29^ show decreased rates similar to those observed previously for Rab3a [9]. In addition, we have solved the crystal structures of Sec4p^V29^ in the GDP-bound state and in complex with Sec2p in order to analyze the structural properties of the mutant. The structural refinement for the complex Sec4p^V29^.Sec2p reveals an intermediate state of the nucleotide dissociation reaction with the GDP bound to Sec4p that we postulate is the result of the reduced rate of GDP release from the mutant V29. We have also performed nucleotide exchange reactions using different truncations of Sec2p in order to determine the minimal region of Sec2p necessary for its GEF activity. We found that amino acids 142–160 of Sec2p are essential for full exchange activity, despite their distal location from Sec4p binding region. Even after much study, many aspects of Rab function remain enigmatic in terms of the structural mechanisms used for their signaling output. A complete understanding of the structural properties of Sec4p, together with functional analysis of interactions with Sec2p and other regulators provides important insights into the structure/function relationships of Rab proteins during the transport process.

## Methods

### Cloning expression and purification

The proteins used in this study were NH_2_-terminally fused to a 6xHis-SUMO tag unless otherwise stated (Table 1). All DNA sequences for *SEC4* and *SEC2* were cloned using BamHI and XhoI restriction enzymes and were expressed recombinantly in *E. coli* strain BL21 (DE3). Cells were cultured at 37 °C until reaching an OD_600nm_ of 0.6 followed by induction of protein expression using 50 μM IPTG and incubation at 15 °C for 14 h. Cells were harvested at 4000 × *g* for 30 min and resuspended in a lysis buffer containing 20 mM Tris-HCl pH 8.0, 300 mM NaCl, 5 mM MgCl_2_. The cells were lysed with 5 cycles of sonication while chilled in an ice bath. Unbroken cells and debris were pelleted at 18,000 × *g* for 30 min at 4 °C and supernatant solution was used for protein purification. The first round of purification was achieved by incubating the lysate with cobalt-conjugated beads (GoldBio Technologies). The protein was eluted in presence of buffer containing 20 mM Tris-HCl pH 8.0, 300 mM NaCl, 5 mM MgCl_2_ and 300 mM imidazole. The sumo tag was cleaved by adding SUMO protease to the eluted sample and the protein mixture was dialyzed against lysis buffer for 12 h at 4 °C. The digested sample was incubated with cobalt-conjugated beads and the unbound fraction containing the protein of interest was recovered. As a final purification step, the protein sample was concentrated and applied to a size exclusion column.

**Table 1:** Bacterial strains used in this study

Strain number	Bacterial strain	Description	Vector used
RCB4554	BL21 (DE3)	Sec4p^V29^ (19–187)	ppSUMO
RCB4699	BL21 (DE3)	Sec4p^WT^ (19–187)	ppSUMO
RCB4636	BL21 (DE3)	Sec2p (51–142)	ppSUMO
RCB4727	BL21 (DE3)	Sec2p (51–182)	ppSUMO
RCB5290	BL21 (DE3)	Sec2p (51–160)	ppSUMO
RCB5291	BL21 (DE3)	Sec2 (1–160)	ppSUMO
RCB5117	DH5α	Sec2p (51–182) mutant 153–157Ala	ppSUMO
RCB5119	DH5α	Sec2p (51–182) mutant 142–147Ala	ppSUMO
RCB5121	DH5α	Sec2p (51–182) mutant F109A	ppSUMO
RCB5214	BL21 (DE3)	Sec4p (19–187) mutant E80A and R81A,	ppSUMO
RCB5215	BL21 (DE3)	Sec2p (51–142) K140C,	ppSUMO

### Preparation of Sec4p.GDP.Sec2p complex

Sec4p_19 − 187_^*S*29*V*^ and Sec2p_51–142_ were first purified separately and then a 1.8-fold molar excess of Sec2p_51–142_ was mixed with Sec4p_19 − 187_^*S*29*V*^ in buffer containing 20 mM Tris-HCl pH8.0 100 NaCl, 5 mM MgCl_2_ for 2 h at 4 °C. After incubation, the protein mixture was concentrated and applied into a size exclusion column Superdex200 (10/300) (GE Healthcare) to isolate the protein complex. Complex formation was evaluated by SDS-PAGE.

### Crystallization and data collection

Crystallization trials for Sec4p_19 − 187_^*S*29*V*^ were performed at a protein concentration of 20 mg/ml. After initial crystal screening small crystals were obtained in presence of 14 % PEG3350 and 200 mM Zinc Acetate. After refinement of the initial condition, large single crystals were obtained in 14 % PEG4000, 50 mM Zinc Acetate with the addition of 10 mM GDP to the drop. Crystals were grown by the hanging-drop vapor-diffusion method after incubation at 20 °C for 10 days. Crystals were soaked in a cryoprotectant solution containing the mother liquor (14 % PEG4000, 50 mM Zinc Acetate) supplemented with 20 % glycerol prior to flash-cooling in 100 K nitrogen gas stream. Diffraction data were collected in a rotary anode with crystal diffracting up to 1.9 Å resolution. The crystal belongs to space group P2_1_, and Matthews’s coefficient calculation [18] indicated the presence of two monomers in the asymmetric unit, giving a solvent content of 40 %. Data collection statistics are summarized in Table 2.

**Table 2:** Data collection and refinement statistics

	Sec4p^V29^	Sec4p^V29^.Sec2p complex
	Data collection statistics	
Space group	P2_1_	I222
Unit cell		116.900 119.280 122.860
a, b, c (Å)	31.720, 75.450, 66.230	
β (°)	91.58	
Wavelength (Å)	1.54180	1.08090
Resolution range (Å)	28.30–1.90 (2.00-1.90)	60.00–2.90 (2.90–3.06)
No. reflections/no. unique reflections	39912/23244	152363/19373
Redundancy	1.7 (1.6)	7.9 (8.1)
Completeness (%)	94.8 (98.3)	99.8 (100)
R_meas_ (%)	7.1 (38.9)	9.0 (54.5)
I/σ (I)	9.1 (2.3)	5.1 (1.5)
	Refinement statistics	
Resolution range (Å)	28.30–1.90	59.64–2.90
no. of reflections (working data/test data)	21713/1170	17371/879
R-factor/R-free (%)	23.9/29.7	25.8/29.5
Protein atoms	2598	2867
Water molecules	464	16
Mean B value/Wilson B value (protein, all atoms) (Å^2^)	17.2/22.0	51.5/66.8
Mean B value (ligand) (Å^2^)	16.3	74.2
rmsd bond length (Å)	0.011	0.012
rmsd bond angle (°)	1.432	1.613

Crystallization trials for the ternary complex Sec4p_19 − 187_^*S*29*V*^.GDP.Sec2p_51–142_ were performed at a protein concentration of 15 mg/ml. Crystals were grown by mixing protein complex with a reservoir solution containing 24 % PEG3350 0.2 M Sodium Citrate in a 1:1 ratio and with the addition of 10 mM GDP to the drop. Crystals were soaked in a cryoprotectant solution containing the mother liquor (14 % PEG4000, 50 mM Zinc Acetate) supplemented with 20 % glycerol prior to flash-cooling in 100 K nitrogen gas stream. The crystallographic data was collected at the Brookhaven National Laboratories (BNL) National Synchrotron Light Source (NSLS). The crystal diffracted up to 2.9 Å resolution and belong to space group I222 with one ternary complex per asymmetric unit (% solvent).

### Structural determination and refinement

All the data sets were processed using Mosflm [19] and Scala [20, 21]. The crystal structure for the mutant S29V of Sec4p was solved by molecular replacement with the program Phaser [22] using the wild-type structure of Sec4p.GDP as a model (accession code: 1G16) [8]. The crystal structure for the ternary complex Sec4p_19 − 187_^*S*29*V*^.GDP.Sec2p_51–142_ was also solved with Phaser [22] using a previously solved structure of the complex Sec4p.Sec2p bound to a phosphate molecule (accession code: 2EQB) [17]. The structure refinement was performed by alternating automatic refinement using Refmac [23], Phenix [24] and manual inspection using Coot [25].

### Preparation of Sec4p.mantGDP and enzyme assays

In order to perform the nucleotide exchange experiments all the GTPases used in this work were preloaded with mantGDP. The procedure consists of incubating the protein sample (buffer: 20 mM Tris–HCl pH8.0 100 mM NaCl, 2 mM MgCl_2_) with a 2-fold excess of mantGDP over the protein concentration and 5 mM EDTA. The mixture was incubated at room temperature for 10 min and the reaction stopped with the addition of 10 mM MgCl_2_. The unbound nucleotide was separated from the sample using a spin column (Micro Bio-Spin 6, Bio-Rad) and the protein eluted in buffer containing 20 mM Tris-HCl pH8.0, 100 mM NaCl, 5 mM MgCl_2_. The protein concentration was measured by Bradford [26].

Fluorescence measurements were performed in buffer containing 20 mM Tris–HCl pH8.0 100 mM NaCl, 5 mM MgCl_2_ at room temperature. All the measurements were carried out in a QuantaMaster™ 40 fluorescence spectrometer (PTI). The mantGDP was excited at 360 nm and emission detected at 450 nm. For the intrinsic nucleotide exchange assay, time-based fluorescence was performed by collecting the fluorescence intensity in intervals specified in the plots. The data were normalized and plotted using Kaleidograph software (Synergy Software).

### Fast kinetics

The fast kinetic experiments were performed in buffer containing 20 mM Tris–HCl pH8.0 100 mM NaCl, 5 mM MgCl_2_ at room temperature. The experiments were carried out using a stop flow apparatus coupled to a QuantaMaster™ 40 fluorescence spectrometer (PTI). The mantGDP was excited at 360 nm and emission was detected at 450 nm. The data were normalized and plotted using Kaleidograph software (Synergy Software).

### *In vivo* assay for Sec2p function

In order to assess if various Sec2 constructs could act as the only copy of Sec2 in the cell transformants of a Sec2 tester strain were streaked to 5-FOA media to select for loss of the *URA3 SEC2* plasmid. Yeast expressing wild type *SEC2* can survive equivalently on the 5-FOA containing media, while a control plasmid with no insert cannot.

### Availability of supporting data

The data set supporting the results of this article are available in the Protein Data Bank (PDB) repository Accession Codes 4ZDW and 4Z8Y.

## Results

### Sec4p^V29^ is only partially activated by Sec2p

**Fig. 2:**
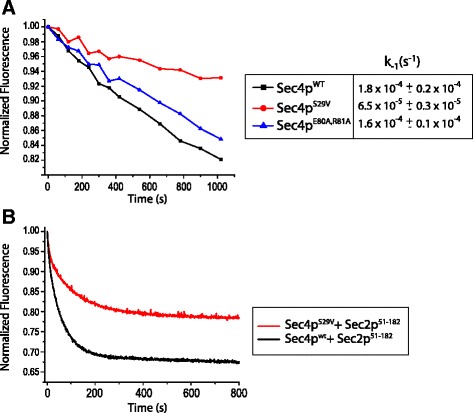
Sec4p^V29^ shows decrease sensitivity to the activation by Sec2p. **a** Intrinsic nucleotide dissociation in presence of 0.3 mM GDP. 0.3 μM of either Sec4p^wt^ (*black*), Sec4p^V29^ (*red*) or Sec4p^E80A,R81A^ (*blue*) preloaded with mantGDP was excited at 360 nm and the emission signal was detected at 450 nm. The assays were done at room temperature with buffer containing 20 mM Tris HCl pH 7.5, 100 mM NaCl and 5 mM MgCl_2_. The plots represent one of the three measurements acquired. **b** mantGDP dissociation from 0.3 μM of either Sec4p^wt^ (*black*) or Sec4p^V29^ (*red*) was measured by the reduction in fluorescence intensity after mixing excess of unlabelled GDP (300 μM) plus 66nM Sec2p_51–182_. Assays were performed at 16 °C with buffer containing 20 mM Tris HCl pH 7.5, 100 mM NaCl and 5 mM MgCl_2_

To analyze the differences in activation between Sec4p^wt^ and Sec4p^V29^ we performed nucleotide dissociation experiments using standard procedures [27–29] where Sec4p is first preloaded with mantGDP nucleotide and then incubated with excess of unlabeled GDP in the presence or absence of Sec2p. To minimize the influence of photo-bleaching in the intrinsic nucleotide dissociation experiments (in the absence of Sec2p), the fluorescence was measured in discrete intervals with the shutter closed between each measurement. The intrinsic nucleotide dissociation rate of mantGDP from the mutant Sec4p^V29^ was observed to be 6.5 × 10^−5^ s^−1^ (Fig. 2a). The single substitution of residue S29 to valine decreases the intrinsic nucleotide dissociation of Sec4p by almost 3-fold in comparison with the wild type form of the protein (1.8 10^−4^ s^−1^). The rates obtained for the intrinsic nucleotide dissociation of Sec4p were very similar to those calculated for Rab3a [9]. The mutant S31V of Rab3a presents a nucleotide dissociation rate of 5.0 × 10^−5^ s^−1^ which is 4 fold slower than the 2.2 × 10^−4^ s^−1^ calculated for the wild type protein [9]. We also compared the nucleotide dissociation from Sec4p when stimulated by its GEF Sec2p (Fig. 2b). The data shows that Sec2p stimulates nucleotide dissociation of Sec4p^V29^ at a significantly slower rate. In addition, the two curves present a noticeable difference in amplitude, which we interpret as being a result of partial dissociation of mantGDP nucleotide from Sec4p^V29^. Previous work has shown that Sec2p stably associates with the GDP bound form of Sec4p^V29^ but exclusively with the free form of Sec4p^wt 30^. We hypothesized that Sec2p is able to bind and change the conformation of both switch regions of Sec4p^V29^ in a similar fashion to that of the wild type protein (confirmed by the ternary complex Sec4p^V29^.GDP.Sec2p). However the bound nucleotide does not completely dissociate from its complex with Sec4p^V29^, which could be explained by the decrease in the nucleotide dissociation observed in our experiments (Fig. 2a). To evaluate the structural consequences of the mutation S29V on Sec4p and how it affects the interaction with Sec2p, we solved the structure of Sec4p^V29^.GDP in presence and absence of Sec2p.

### Crystal structure of Sec4p^V29^

**Fig. 3:**
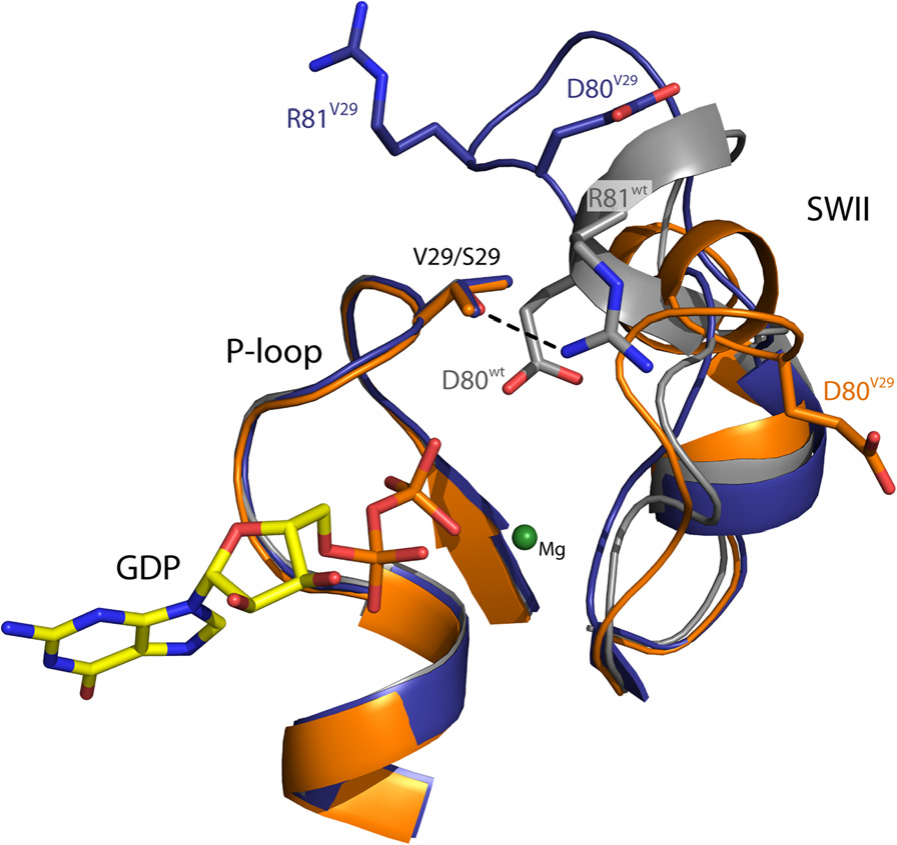
Superposition of the P-loop and SWII region between molecule A of Sec4p^wt^ (*grey*) (6) and molecule (**a**) (*orange*) and (**b**) (*purple*) of Sec4p^V29^

Crystals for the mutant Sec4p^V29^ were obtained and the structure was solved at 1.9 Å resolution using the wild type Sec4p bound to GDP as a model for molecular replacement. After several rounds of refinement the final model converged to an R_free_ of 29 % and R_work_ of 23 %. As expected the overall structure of Sec4p^V29^.GDP is almost identical to the Sec4p^wt^.GDP (PDB:1G16) not considering the switch regions (RMSD 1.2 Å for 159 Cα atoms). Some of the crystal contacts are made through interactions with zinc atoms that are coordinated by Asp43 and Asp166 from molecule B, and His121 from molecule A. Besides the presence of the mutation S29V, the P-loop region from Sec4p^V29^.GDP and Sec4p^wt^.GDP [8] superpose almost perfectly. Furthermore, all the interactions between protein and nucleotide are conserved between the two structures. The structure Sec4p^V29^.GDP contains two molecules in the asymmetric unit (chains A and B). The switch I region is disordered in the molecule A and ordered in molecule B. The SWI of molecule B adopts a different conformation from that observed on the Sec4p^wt^.GDP structure. However, this new conformation is held in place by interactions with symmetric related molecules and is probably an artifact of crystal packing. The switch II region for molecule A presents the same conformation observed on molecule C of the Sec4p^wt^.GDP structure [8]. In this conformation the Gly78 is within 4 Å from the Ser29 (WT) or Val29 (mutant). In contrast, the molecule B presents a dramatic change in conformation that pulls the Gly78 from the close contact with Val29 and the active site at a distance of almost 8 Å. However, the region strongly interacts with symmetric related molecules and its conformation is probably an artifact of crystal packing. One of the most intriguing observations made for the Sec4p^wt^.GDP was the interaction between the Arg81 of the SWII and the Ser29 of the P-loop. The side chain of the residue Arg81 forms a 2.8 Å length hydrogen bond with the side chain of the Ser29 residue. This interaction is not observed in the Sec4p^V29^.GDP structure (Fig. 3). To analyze if the loss of this interaction could somehow explain the decrease in nucleotide dissociation, we reasoned that mutating the residue Arg81 in the wild-type protein would eliminate this interaction and the intrinsic rate could be measured and compared to the S29V mutant. Together with the Arg81 we have also mutated the residue Glu80, since this residue is very close to the Ser29 and could compensate the lack of the Arg81. We then measured the intrinsic dissociation of mantGDP from the double mutant protein (E80A, R81A). The double mutant shows a slight decrease in the intrinsic dissociation of nucleotide compared to the wild type. However, the decrease was not to the same extent as observed for the S29V protein.

Our data shows that the mutation S29V on Sec4p is responsible for the reduction in both intrinsic and stimulated nucleotide dissociation. This mutation does not appear to cause any gross conformational differences in the three-dimensional structure of the protein. Therefore the increased affinity between Sec4p^V29^ and the nucleotide is probably due to loss of intramolecular electrostatic interactions caused by the presence of the valine non-polar side chain. A similar issue has been observed previously for the oncogenic mutant of Ras GTPase.

### Crystal structure of the ternary complex Sec4p^V29^.GDP.Sec2p

**Fig. 4:**
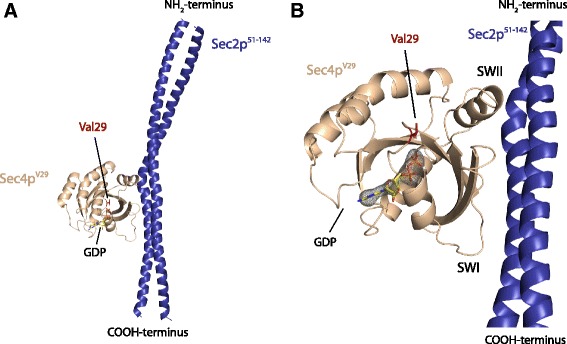
Crystal structure of the ternary complex Sec4p^V29^.GDP.Sec2p. **a** Overall structure of the complex showing the presence of the GDP molecule bound in the active site. **b** Fo - Fc map contoured at 3σ (*grey*) indicates the presence of the nucleotide

**Fig. 5:**
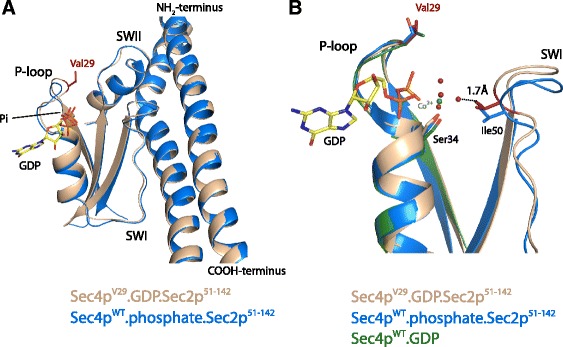
**a** Superposition between Sec4p^V29^.GDP.Sec2p and Sec4p^WT^.phosphate.Sec2p using the Cα atoms of the Sec4p binding site of Sec2p (residues 100–120). The V29 mutant shows a slight shift in the active site. The P-loop, nucleotide and other regions of the protein shift for about 1 Å in the direction of Sec2p. This shift causes a slightly more closed conformation in comparison with the wild type complex Sec4p^WT^.phosphate.Sec2p. **b** Superposition between Sec4p^V29^.GDP.Sec2p and Sec4p^WT^.phosphate.Sec2p performed using the P-loop (Cα residues 26–33) of Sec4p. In this superposition the residue Ile50 of Sec4p^V29^ shifts toward the active site, coming very close to one of the waters that coordinate the magnesium ion. Waters are represented by red spheres. Residues that participate on the magnesium coordination are shown

The structural and functional mechanism by which the GEF Sec2p activates Sec4p has been addressed previously [5, 7, 27]. The nucleotide exchange mechanism follows the classic allosteric competitive model in which Sec2p is able to bind to the complex Sec4p.GDP at much lower affinity in comparison to Sec4p free from nucleotide [27, 30, 31]. After binding, Sec2p extensively changes the conformation of switch I and switch II leading to the release of magnesium and consequently the decrease of the Sec4p affinity for nucleotide [5, 7]. In the absence of nucleotide the affinity of Sec4p and Sec2p is significantly increased [27]. Previous work has suggested that Sec4p^V29^ is able to stably associate with Sec2p in the GDP bound state [30]. However, the role of the residue Ser29 during Rab activation is not well understood. In our experiments we were able to confirm this observation by forming a stable complex between Sec4p^V29^ and Sec2p_51–142_ in presence of nucleotide and magnesium (data not shown). The wild type Sec4p can only stably associate with Sec2p in the absence of magnesium [5]. A common procedure to deplete nucleotide in order to obtain a GTPase in complex with its respective GEF is the addition of EDTA to purification buffers [5, 32–34]. In presence of nucleotide and magnesium the Sec4p.Sec2p complex tends to dissociate, reflecting the low affinity (73 μM) between Sec2p and the GDP bound form of Sec4p [27]. In contrast, the addition of GDP and magnesium does not disrupt the complex between Sec4p^V29^ and Sec2p. This enabled us to obtain crystals for the ternary complex Sec4p^V29^.GDP.Sec2p and solve the structure at 2.9 Å resolution (Fig. 4a). As seen in the other two structures of the complex Sec4p.Sec2p the average temperature factor is very high (66.8 Å^2^) which reflects the high flexibility of the complex [5, 17]. Besides the alpha and beta phosphates of the GDP that present temperature factors similar to the overall average, the rest of the molecule presents very high flexibility. However, the presence of the nucleotide is confirmed by the OMIT map at 3σ (Fig. 4b). Superposition with previously solved structures of the complex Sec4p.Sec2p reveals that Sec4p^V29^.GDP.Sec2p shares high similarity with the Sec4p.Sec2p complex bound to phosphate [5] with a RMSD between the 353 Cα atoms of 0.675 Å. However, due to the presence of the guanine ring the loop β6-α5 adopts a conformation that resembles the Sec4p.GDP and Sec4p.GTP structures. The S29V mutation does not directly affect the interface of interaction with Sec2p but, as our data suggests, it affects the nucleotide release (Fig. 2b). When the superposition between Sec4p^V29^.GDP.Sec2p and Sec4p.Phosphate.Sec2p structures is performed using only the Cα atoms of the Sec4p binding site of Sec2p (residues 100–120), it is clear that the mutant active site is in a more closed conformation than the wild type (Fig. 5a). Both the P-loop and the nucleotide are shifted in about 1 Å towards the SWI region in the V29 structure (Fig. 5a). However, it is difficult to predict if this conformational difference in the Sec4p^V29^.GDP.Sec2p could cause the decreased nucleotide dissociation rates. The superposition of the P-loop of Sec4p^V29^.GDP from the complex with Sec2p with the P-loop of Sec4p.GDP/GTP reveals that one of the water molecules that coordinate the Mg^2+^ ion is only about 1.7 Å distance from the Ile50 (Fig. 5b). This residue is known for its involvement in the nucleotide exchange reaction by assisting magnesium dissociation and blocking magnesium rebinding [5]. The shift observed in both P-loop and nucleotide in our structure could interfere with the rebinding of magnesium possibly causing the decreased rates measured for nucleotide exchange.

### Structural and functional analysis of the coiled coil domain of Sec2p

**Fig. 6:**
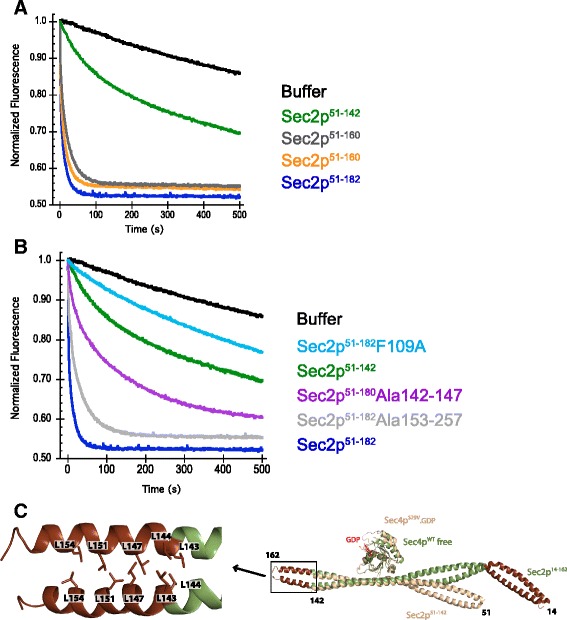
Sec2p truncations affecting nucleotide dissociation activity. MantGDP dissociation from Sec4p was measured by reduction in fluorescence intensity after mixing excess of unlabeled GDP (300 μM) plus: buffer (black) or 500nM of different truncations of Sec2p (**a**). Analysis of how mutations on amino acid region 142–160 of Sec2p affect the stimulation of nucleotide dissociation of Sec4p are shown on (**b**). All assays were done using 1 μM Sec4p_19–187_ preloaded with mantGDP. The superposition between the complexes Sec4p.GDP.Sec2p and Sec4p.Sec2p is shown on (**c**). Regions of Sec2p that were truncated for the crystallization of Sec4p.GDP.Sec2p complex are colored in brown in the Sec2p structure of the complex Sec4p.Sec2p (reference). Leucine residues suggested to be important for the GEF activity of Sec2p are labeled

**Fig. 7:**
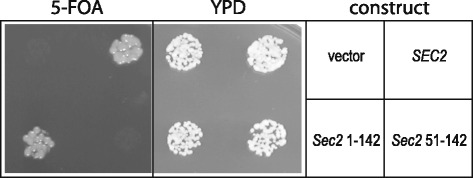
Sec2 residues 1–142 are sufficient and necessary for cell viability; a construct containing this region of Sec2 can provide the sole source of *SEC2* function in a tester strain on 5-FOA media. Truncation of the first fifty residues abolishes this effect, which is evident in the absence of colonies on the 5-FOA media in the Sec2 51–142 construct. Vector only and *SEC2* wild type constructs are shown for reference

In spite of the rather fast intrinsic nucleotide dissociation rate of Sec4p its GEF Sec2p can still stimulate nucleotide dissociation [27]. Sec2p binds to Sec4p as a homo-dimer composed of a very long coiled coil (160 residues) in a region that is situated in the NH_2_-terminal of Sec2p. As discussed previously the binding site of Sec4p is located within the central region of the coiled coil between the residues 100 and 120. Important residues for this interface have been previously mutated and the stimulation of the dissociation rate is known to be affected by mutations in this region [17]. These the biochemical assays together with the crystal structures for the complex have defined the minimal region of Sec2p necessary for the binding to Sec4p. However, the minimal region of Sec2p that still retains full GEF properties has not been yet described. We performed a series of truncations on the coiled coil domain of Sec2p and analyzed its nucleotide dissociation activity. We avoided using truncations of Sec2p that are known to show impaired binding to Sec4p [17]. The standard truncation of Sec2p that has been largely used for kinetic assays contains the region between residues 1–160 [5, 17, 27]. Our data shows that the truncation Sec2p^51–160^ contains similar activity when compared to Sec2p^1–160^, which demonstrates that the first 50 residues of Sec2p are dispensable for its GEF activity (Fig. 6). In addition, the truncation of Sec2p^51–182^ containing 22 extra amino acids in the COOH-terminal of the predicted coiled coil domain did not alter activity. Surprisingly, however, the truncation Sec2p_51–142_ showed a dramatic decrease in the nucleotide exchange stimulation of Sec4p (Fig. 6a). The dissociation rate for the truncation Sec2p_51–142_ is 20 fold slower than the rate calculated for the truncation Sec2p^1–160^. Although this truncation drastically affects activity it is still able to form a stable complex with Sec4p as attested by the crystal structure of the complex (Fig. 4a). The residue Phe109 of Sec2p is known to actively participate in the complex interface with Sec4p. The truncation 51–142 does not affect the Sec4p binding site but yet decreases the protein activity close to the level of the mutant F109A of Sec2p. In order to investigate what causes this large decrease in activity observed we analyzed the region between the residues 142–160 on the crystal structure for Sec4p.Sec2p free from nucleotide [5]. As exemplified on Fig. 6c this region is characterized by the presence of a canonical coiled coil where several leucine residues (Fig. 6c) participate in the “knobs-into-holes” packing characteristic of coiled coils [35]. To investigate this problem we performed alanine scanning mutagenesis on the truncation Sec2p^51–182^. Two constructs were designed where alanines replaced the residues 142–147 (TLLDTL) and 153–157 (NLKKV). In the first construct alanines substituted a total of three leucine residues (Leu143, Leu144 and Leu147), two of which participate in the coiled-coil packing. In the second construct only the Leu154 participates in the coiled coil formation. As expected the mutant Sec2p^Ala142–147^ shows a large reduction in the nucleotide dissociation with a rate similar to the truncation Sec2p_51–142_ (Fig. 6b). For the mutant Sec2p^Ala153–157^ the dissociation rate is less affected, but also shows a slight reduction in activity. The alanine scanning data suggests that the region between residues 142–160 which is part of the coiled coil domain of Sec2p is crucial for GEF activity. Disruption of key coiled coil packing residues in both constructs substantially decreases activity of Sec2p_51–182_ almost to the same level of Sec2p^51–142^. Therefore, we can predict that the disruption of all residues important in the coiled coil packing for this region would bring the level of dissociation rate of the Sec2p_51–182_ to the level of the truncation Sec2p_51–142_. These data suggest that the region between amino acids 142–160, a region which engages in classical coiled coil packing does not affect binding to Sec4p, but nevertheless it is still crucial for the GEF activity of Sec2p. Interestingly, while the truncation Sec2p^1–142^ is functional *in vivo*, cells containing Sec2p^51–142^ are not viable (Fig. 7). These data indicates that the first 50 residues are important for a still unappreciated part of Sec2p function.

### Enhancement of dimerization with disulfide bond increases activity of the truncation Sec2p_51–142_

**Fig. 8:**
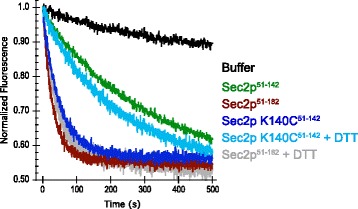
Disulfide bond introduction between Sec2p monomers increases protein activity. MantGDP dissociation from Sec4p was measured by reduction in fluorescence intensity after mixing excess of unlabeled GDP (300 μM) plus: buffer or 250nM of different Sec2p constructs. The mutant K140C increases the rather low dissociation rate Sec2p_51–142_. When DTT (50 mM) is added to the buffer solution the dissociation level drops close to the wild type level. The addition of DTT to the truncation Sec2p_51–182_ is not deleterious to the protein activity. All assays were performed with 1 μM Sec4p_19–187_ preloaded with mantGDP

We have shown previously that the truncation Sec2p_51–142_ presents a large reduction in activity caused by the absence of the region 142–160. However, the reason this region is so important for Sec2p activity is not clear. We analyzed two possible hypotheses. First, the presence of this region might apply constrains on the COOH-terminal of the coiled coil helices that could be carried through the binding site and interaction with Sec4p. In this case we should observe differences in the Sec2p coiled coil in the region downstream to the Sec4p binding site of Sec2p when the Sec4p molecule in the structures of Sec4p^V29^.GDP.Sec2p_51–142_ and Sec4p.free.Sec2p_14–165_ are superposed (Cα residues 100–120). Besides conformational differences observed by the COOH-terminal of Sec2p, there are no major differences in the interaction interface between Sec4p and Sec2p between both structures. The second hypothesis is based on the fact that Sec2p forms a dimer and the GEF activity depends on the dimerization of the two monomeric helices. We could expect that disturbing the dimer formation of Sec2p would lead to loss of GEF activity. Since the region 142–160 presents an important dimerization interface, we hypothesized that the deletion of this region as seen in the truncation Sec2_51–142_ would lead to a weaker interaction between the two monomers of Sec2p, directly reflecting a decrease in the dissociation constant for the dimer. To address this question we designed mutants in which a cysteine residue from one monomer would be able to interact with the symmetric residue from the other monomer and form a disulfide bond to generate a stable dimer. We designed two mutants, a single mutant on the COOH-terminal of Sec2p_51–142_ (K140C) and a double mutant where both upstream and downstream regions of the Sec4p binding site were mutated (A91C and K140C). The double mutant of Sec2p generated insoluble protein and was not used in further experiments. The single mutant Sec2_S1 ‐ 142_^K140C^ rendered a very soluble and stable protein and was used for the nucleotide dissociation assays. The mutation K140C dramatically increased the GEF activity of the truncation Sec2p_51–142_ (Fig. 8). The dissociation rate of Sec2_S1 ‐ 142_^K140C^ mutant is very similar to the truncation containing the region 142–160 of Sec2p (Sec2p_51–182_, Sec2p_51–160_ or Sec2p_1–160_). These data suggest that the formation of a disulfide bond between the two monomers of Sec2p increases the affinity constant and the increase in protein activity reflects enhanced dimer stability. Treatment with a reducing agent (DTT) brings the activity of the mutant Sec2_S1 ‐ 142_^K140C^ back close to its normal levels observed with Sec2p_51–182_ (Fig. 5). The amount of DTT used for the experiments was rather high (50 mM); to evaluate for any possible deleterious effects on dissociation rate due to the reducing agent, the highly active truncation Sec2p_51–182_ was tested as a control. DTT does not affect the dissociation rate of Sec2p_51–182_, but drastically reduces the activity of Sec2_S1 ‐ 142_^K140C^ Altogether, these data suggest that the coiled coil region 142–160 of Sec2p positively influences protein activity and the reason for this increase is due to an increase in the affinity of dimer formation.

## Discussion and Conclusions

In this work we have investigated the nucleotide exchange mechanism of the Rab GTPase Sec4p and how its GEF Sec2p stimulates nucleotide dissociation, making use of the Sec4p mutant S29V corresponding to Gly12 in Ras proteins. Ras Glycine 12 has been extensively linked to the formation of cancer cells caused by the imbalance in the GTPase cycle of Ras. Since mutations on the same residue in Rab GTPases have been suggested to display the opposite phenotype, mutating the Ser29 to Val in Sec4p offered us a tool to study Sec4p activation giving us insights into the basic differences between members of the Ras GTPase superfamily.

We have determined that the mutation S29V clearly reduces the intrinsic nucleotide dissociation rate of Sec4p in comparison to the wild type protein. In order to investigate the structural implications of the mutation S29V we solved the structure of Sec4p^V29^ in the GDP bound state. The structure of Sec4p^V29^.GDP presents a very similar fold to the wild type protein indicating that the decrease in the intrinsic nucleotide dissociation rate is not due to large conformational changes. This is similar to observations of equivalent mutations in other GTPase family members. The structures of the mutant G12V of p21^ras^ and Cdc42 for example do not exhibit explicit conformational changes due to the G12V mutation when compared with the wild type equivalents [36–39].

The difference in nucleotide dissociation rate is most likely explained by changes in the protein-nucleotide environment created by the hydrophobic side chain of the Val29. The mutation from the polar residue serine to the just slightly larger hydrophobic valine creates a more hydrophobic local environment. We can predict that the energy cost to solvate the active site of the mutant Val29 would be larger than for the wild type protein and could indirectly result in the increased binding affinity of the protein for the nucleotide. Furthermore, Sec2p stimulates the nucleotide dissociation from Sec4p^V29^ at a slower rate than the rate observed for Sec4p^wt^. During activation Sec2p binds to Sec4p causing conformational changes leading to the loss of magnesium and decreased affinity for the nucleotide. For the mutant, an increase in the affinity for the nucleotide due to the presence of Val29 would decelerate nucleotide release and explain the observed decrease in the intrinsic nucleotide dissociation rate. Another interesting property of the mutant is that Sec2p can stably associate with the GDP bound state of Sec4p^V29^ even in the presence of magnesium. In contrast, Sec2p preferentially associates with the nucleotide free state of wild type Sec4p. We analyzed the crystal structure for the complex Sec4p^V29^ and Sec2p and determined that the complex was bound to a GDP molecule. This complex most likely represents the intermediate state of the nucleotide dissociation just before nucleotide release. Prior to this study two structures for the complex of Sec4p.Sec2p have been described. These structures represent the free form of the complex [5] and the phosphate bound form of the complex [17], and together explain the nucleotide exchange mechanism in molecular detail. Superposition between the two structures that represent the intermediate state of the reaction (Sec4p^V29^.GDP.Sec2p and Sec4p^wt^.phosphate.Sec2p) reveals that besides the overall similarities, a small shift in the P-loop results in a slightly tighter active site. The distance between the P-loop and Switch I, more specifically between the residues Val29 and Ile50 is shortened in comparison with the structure Sec4p^wt^.phosphate.Sec2p. The slight alteration in the active site, places the Ile50 residue very close to the magnesium binding site. It is thought that the Ile50 residue plays a critical role preventing magnesium rebinding to the protein after complex formation [5]. In the free state of the complex Sec4p.Sec2p, the Ile50 residue is placed within 0.9 Å from the magnesium binding site. The shift in the P-loop observed in our structure would place the whole active site closer to the SWI and more specifically to the Ile50 residue. We can predict that this difference would make magnesium rebinding to the intermediate state of the complex more difficult and together with the increase in the affinity for the nucleotide it could explain why the GDP bound state of Sec4p^V29^ can form a stable complex with Sec2p.

During our nucleotide dissociation studies we discovered that the truncation Sec2p_51–142_ stimulates nucleotide release from Sec4p at a much slower rate than the truncation containing the 18 residues in region 142–160. This was somehow surprising because the Sec4p binding site of Sec2p lies between residues 100–120, and the absence of the region 142–160 would not directly affect the protein protein interaction. However, looking closely, this region possesses several leucine residues forming a classical coiled-coil packing interface that could be important for dimer formation and protein stability. Mutation of the leucine residues participating in the coiled coil interaction to alanines reduced protein activity, most likely through a disruption of the dimer coiled-coil structure.

To further validate the idea that disruption of dimerization causes reduced Sec2p activity, we created the mutant K140C on the truncation of Sec2p_51–142_. This truncation of Sec2p contains all the elements necessary for GEF activity however, has a slower activity than a protein which contains the dimer interface region between residues 142–160. The idea is that the formation of a disulfide linkage between the equivalent cysteine residues from both monomers would recapitulate dimerization in the absence of residues 142–160. The results of the nucleotide exchange assays show that in the absence of reducing agent the nucleotide dissociation rate for Sec2_S1 ‐ 142_^K140C^ increased drastically in comparison to the wild type truncation Sec2p_51–142_, to a level similar to the truncation containing the coiled coil region 142–160 of Sec2p. These data indicates that although the region 142–160 is not directly involved in the binding to Sec4p this region is important for protein dimerization and indirectly affects protein activity. In addition, we determined that the first 50 residues in the Sec2p NH_2_-terminus do not affect GEF activity, but are important for full Sec2p function *in vivo*. This study extends the determination of a minimal GEF region for Sec4 and Sec2p interaction and establishes a minimal region of Sec2p containing the fully active enzyme, which we determined to be between residues 51–160. The intrinsic nucleotide dissociation rate of Sec4p is high relative to other Ras-related GTPases and the biological necessity for the potent GEF activity of Sec2p remains a mystery. A complete understanding of the mechanism of Sec2p action, together with functional analysis and interactions with other protein partners will provide important insights into the role of Sec4p and related Rab GTPases during exocytosis.

## Abbreviations

GTP: Guanosine-5′-triphosphate
GDP: Guanosine-5′-diphosphate
GEF: Guanine nucleotide exchange factor
GAP: GTPase activating protein
P-loop: Phosphate binding loop
SWI: Switch I
SWII: Switch II
IPTG: Isopropyl β-D-1-thiogalactopyranoside
mantGDP: *2′-/3′-O*-(N-Methylanthraniloyl)guanosine-5′-O-diphosphate
EDTA: Ethylenediaminetetraacetic acid
DTT: Dithiothreitol
PDB: Protein Data Bank
